# A tumor microenvironment gene set–Based prognostic signature for non-small-cell lung cancer

**DOI:** 10.3389/fmolb.2022.849108

**Published:** 2022-08-10

**Authors:** Yingsheng Wen, Guangran Guo, Longjun Yang, Lianjuan Chen, Dechang Zhao, Xiaotian He, Rusi Zhang, Zirui Huang, Gongming Wang, Lanjun Zhang

**Affiliations:** ^1^ State Key Laboratory of Oncology in South China, Collaborative Innovation Center for Cancer Medicine, Guangzhou, China; ^2^ Department of Thoracic Surgery, Sun Yat-sen University Cancer Center, Guangzhou, China

**Keywords:** non-small-cell lung cancer, prognosis, tumor microenvironment, gene, survival

## Abstract

**Background:** The tumor microenvironment (TME) is involved in the development and progression of lung carcinomas. A deeper understanding of TME landscape would offer insight into prognostic biomarkers and potential therapeutic targets investigation. To this end, we aimed to identify the TME components of lung cancer and develop a prognostic signature to predict overall survival (OS).

**Methods:** Expression data was retrieved from The Cancer Genome Atlas (TCGA) database and differentially expressed TME-related genes were calculated between tumor and normal tissues. Then nonnegative matrix factorization (NMF) clustering was used to identify two distinct subtypes.

**Results:** Our analysis yielded a gene panel consisting of seven TME-related genes as candidate signature set. With this panel, our model showed that the high-risk group experienced a shorter survival time. This model was further validated by an independent cohort with data from Gene Expression Omnibus (GEO) database (GSE50081 and GSE13213). Additionally, we integrated the clinical factors and risk score to construct a nomogram for predicting prognosis. Our data suggested less immune cells infiltration but more fibroblasts were found in tumor tissues derived from patients at high-risk and those patients exhibited a worse immunotherapy response.

**Conclusion:** The signature set proposed in this work could be an effective model for estimating OS in lung cancer patients. Hopefully analysis of the TME could have the potential to provide novel diagnostic, prognostic and therapeutic opportunities.

## Introduction

Lung cancer is the leading cause of cancer-related morbidity and mortality, and in particular, non-small-cell lung cancer (NSCLC) is the most prevalent form Sung et al. (2021). The treatment of NSCLC includes surgery, chemotherapy, and targeted therapy. Recent advances in lung cancer treatments, such as targeted therapy and immune therapy, have improved clinical outcomes, and some patients have shown satisfactory therapeutic responses (Chen et al., 2014). Immune checkpoint blockade (ICB) is an effective approach that disturbs cancer cell immune surveillance subversion (Ribas and Wolchok, 2018). However, despite its efficacy in some patients, many patients fail to respond to ICB. Therefore, alternative therapeutic investigations have taken other pro-tumorigenic cells including macrophages, endothelial cells and fibroblasts into consideration (Mahoney et al., 2015). Hence, an emerging need for full characterization and an in-depth understanding of the TME has surged. Previous studies have shown that the TME plays an important role in the progression and treatment response of NSCLC (Wood et al., 2014). Therefore, genes which are key to TME would be likely differentially expressed between patients at high risk and for those at low risk. Consequently, these genes would be ideal makers for predicting prognosis and therapy response.

In this study, we systematically analyzed the characteristics of TME-related genes in NSCLC patients using data from TCGA RNA-seq datasets. Then, we performed cluster analysis for NSCLC based on TME-related gene expression signatures and divided them into two different clusters. Furthermore, a TME-related gene model was constructed to predict the prognosis of NSCLC patients.

## Methods

### Data acquisition and processing

The gene expression data of 497 lung cancer tissue samples and 54 normal lung tissue samples and the corresponding clinical information were acquired from The Cancer Genome Atlas (TCGA) database (https://portal.gdc.cancer.gov/). 39 sample was rejected for lack of survival outcome. A Wilcoxon test was used to analyze differentially expressed genes in the TCGA sample using the “limma” package in R (The R Foundation for Statistical Computing, Vienna, Austria) (Ritchie et al., 2015). To identify differentially expressed genes in lung cancer, the cutoff threshold in TCGA was set as |log2-fold change (FC)| ≥1.0, and the false discovery rate (FDR) was set at <0.05. The corresponding clinical information of the patients with lung cancer was collected and used for the subsequent analyses. The external validation cohort consisted of the expression data and the comparative clinical data acquired from the Gene Expression Omnibus (GEO) database (https://www.ncbi.nlm.nih.gov/geo/) (GSE50081, GSE13213). The TCGA and GEO sample ids was showed in Supplementary Material (Supplementary Data S1). TME-related genes were obtained from published studies (Newman et al., 2015; Rooney et al., 2015; Becht et al., 2016; Chifman et al., 2016; Li et al., 2016; Tirosh et al., 2016; Aran et al., 2017), and a total of 4061 genes were included. A flow chart to depict the study design was shown in Supplementary Figure S1.

### Subclasses identification

The microenvironment-related genes obtained were subsequently used in nonnegative matrix factorization (NMF) clustering. We used univariate Cox proportional hazards model to examine the associations between gene expression and overall survival. Genes with false discovery rate (FDR) less than 0.01 were considered to be statistically significant and included in consensus clustering analysis. Specifically, NMF was applied to gene expression matrix A that contained prognostically significant TME-related genes aforementioned (Supplementary Data S2). Matrix A was factorized into two nonnegative matrices W and H (i.e., A ≈ WH). Repeated factorization of matrix A was performed and its outputs were aggregated to obtain consensus clustering of samples. The optimal number of subtypes was selected according to cophenetic, dispersion, and silhouette coefficients (Kim and Park, 2007). Unsupervised NMF clustering methods were performed using the “NMF” package of the R software package on the metadata set, and the best cluster number was chosen as the coexistence correlation coefficient K value 2 (Gaujoux and Seoighe, 2010).

### Prognostic model construction

We used the “survival” package (https://CRAN.R-project.org/package=survival) in R to perform a univariate Cox regression analysis for all differentially expressed microenvironment-related genes and screened for significant candidate genes. Subsequently, the prognostic risk characteristics were assessed using the “glmnet” and “caret” R packages based on the least absolute shrinkage and selection operator (LASSO) method (Simon et al., 2011). Then, a prognostic model containing seven microenvironment-related genes was constructed based on the screened candidate genes. According to the median value of the risk score, the patients with lung cancer were classified into high-risk and low-risk groups. Kaplan-Meier analysis was used to construct a survival curve. Then, a log-rank test was applied to compare the overall survival (OS) of the two subgroups. Thereafter, a receiver operating characteristic (ROC) curve was drawn to evaluate the performance of the prognostic model using the “survivalROC” package in R (Heagerty and Zheng, 2005). According to the patient’s clinical information and risk score, independent prognostic clinical factors were selected by multivariate Cox regression analysis. A nomogram was constructed using the survival rate and “RMS” R package (Núñez et al., 2011), and a correction curve was drawn to evaluate the consistency between the actual and predicted survival rates. Moreover, the concordance index (C index) was calculated. The correlation between the risk score and various clinical factors was analyzed using the “limma” package in R and then visualized by the “ggpubr” package in R (Whitehead et al., 2019).

### The correlation between the risk score and cell types

The abundance of tumor-infiltrating microenvironment cells was calculated using the “MCPcounter” package in R (https://github.com/ebecht/MCPcounter) and then correlation analysis was performed with the risk score using the “corrplot” package in R (https://cran.r-project.org/web/packages/corrplot/vignettes/corrplot-intro.html). In addition, we used the “IMvigor” software package in R to obtain the IMvigor dataset, which helped us study the signature-immunotherapy efficiency relationship.

### Statistical analysis

We used Kaplan–Meier analysis to construct survival curves using the “survival” and “survminer” packages in R and a log rank test to evaluate the significance of differences between the two subgroups (https://CRAN.R-project.org/package=survminer). Univariate and multivariate Cox proportional hazards model were used to analyze the association between subtypes and prognosis with R survival package. Wilcoxon signed-rank tests were performed to explore quantitative variables. Significance was defined as *p* < 0.05. All statistical analyses were performed using R version 4.0.3.

## Results

### Classification of non-small-cell lung cancer based on tumor microenvironment genes

**FIGURE 1:**
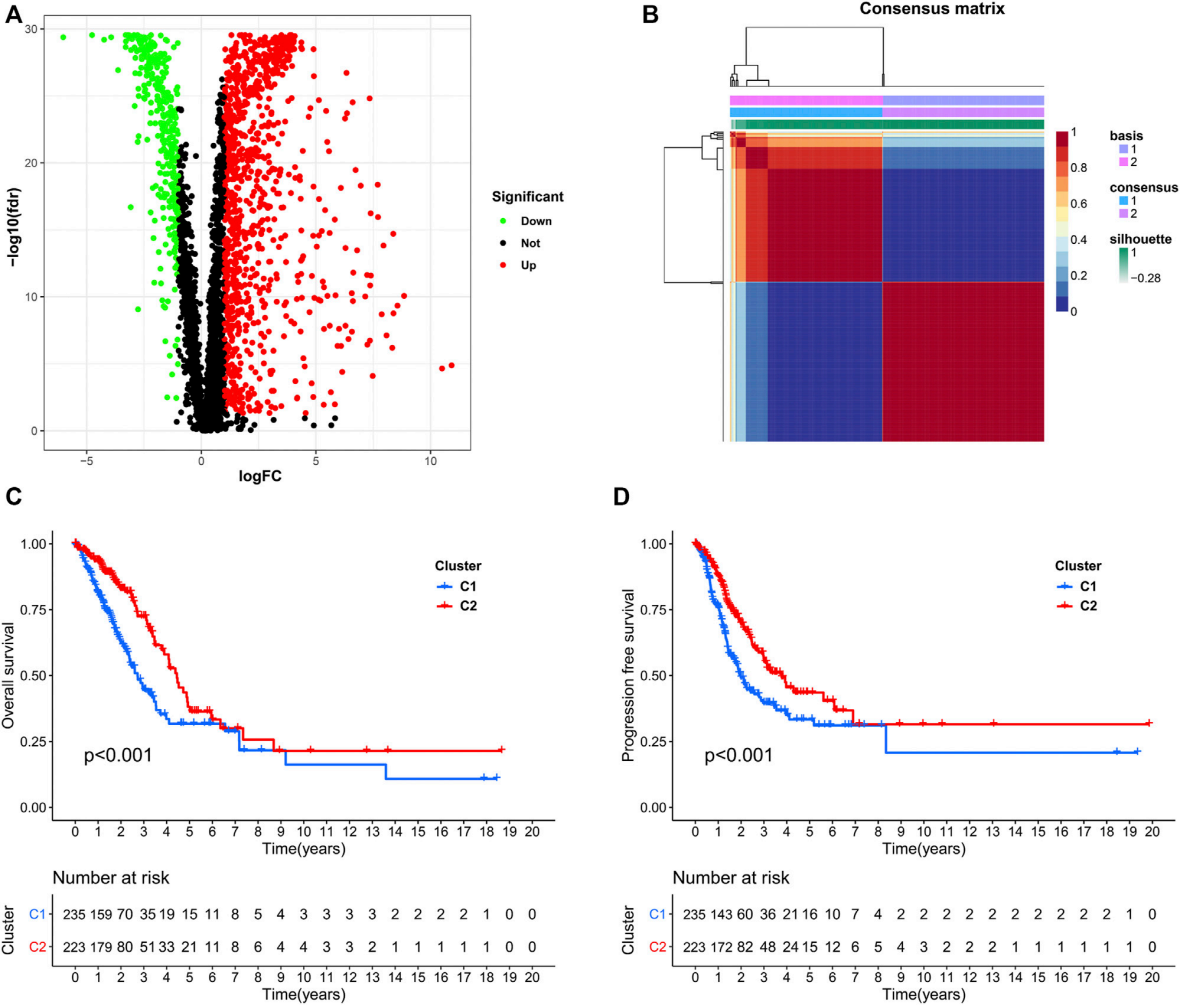
Identification of NSCLC subclasses using NMF consensus clustering. **(A)**, A volcano map of differentially expressed genes related to the TME. **(B)**, NMF clustering using microenvironment-related genes. **(C,D)**, Survival analysis of patients in Clusters one and two.

**FIGURE 2:**
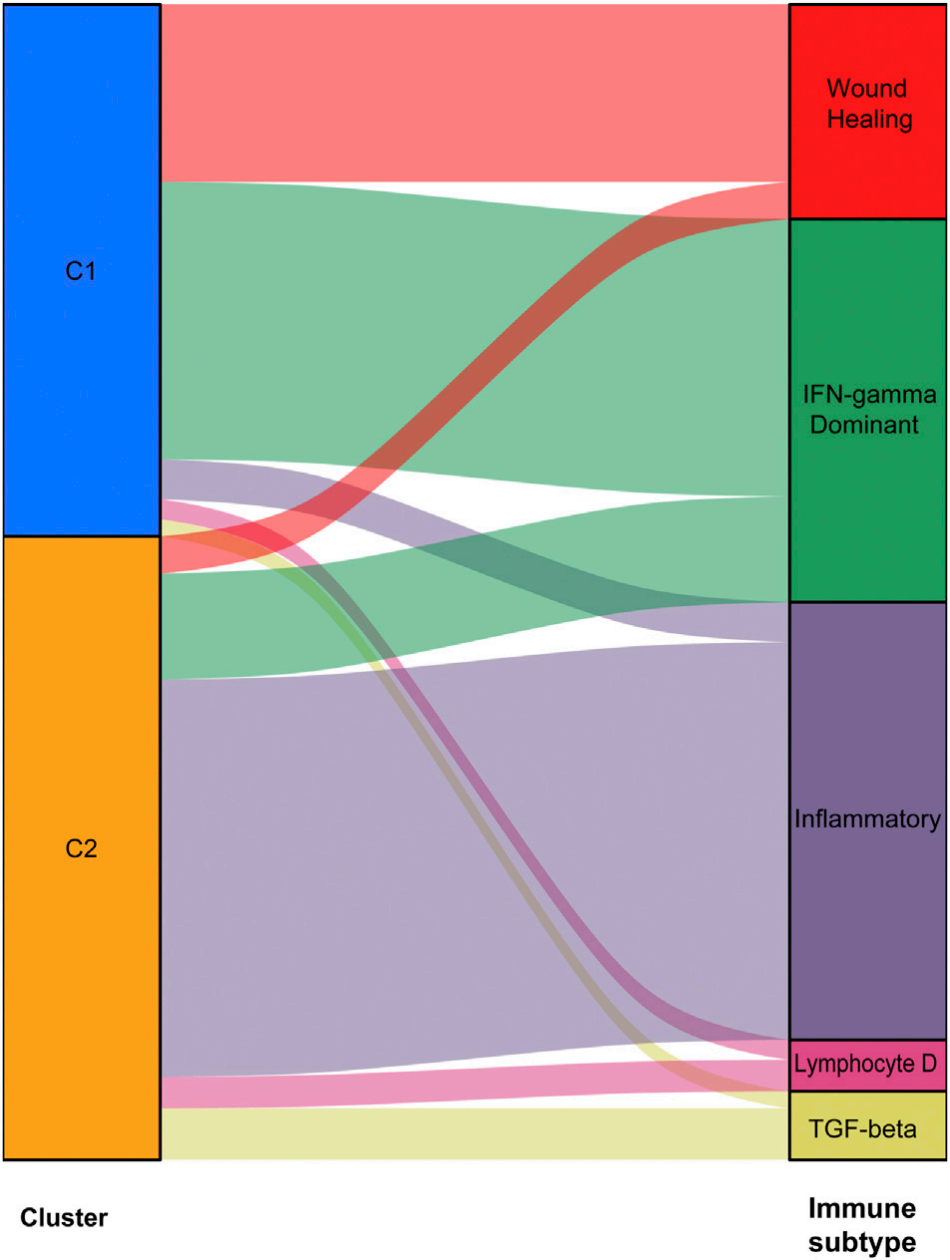
Abundances of immune cell subtypes in two clusters of patients.

We used data from the TCGA dataset to calculate differentially expressed genes between tumor and normal tissues, and genes related to the TME were selected. A total of 1283 differentially expressed genes were identified, of which 949 genes were upregulated in tumor tissues (Figure 1A). Thereafter, NMF analysis was used to divide patients into two different clusters (C1 and C2), where the consensus matrix heatmap maintained a superior boundary (Figure 1B) compared to other classifications with more than two clusters (Supplementary Figure S2). In addition, patients in Cluster two had a longer survival time (Figure 1C) and a better progression survival time (Figure 1D) than Cluster one patients. Considering that immune cells play a key role in the TME, we used a gene expression–based approach to estimate the abundances of specific immune cell types in two clusters of patients. Recent research has identified six immune subtypes associated with the TME and showed that patients with a higher inflammatory subtype signature have the best prognosis. (Thorsson et al., 2018). Consistent with these findings, the inflammatory subtype was also preferentially distributed in Cluster 2 (Figure 2).

### Gene signature for lung cancer prognosis

**FIGURE 3:**
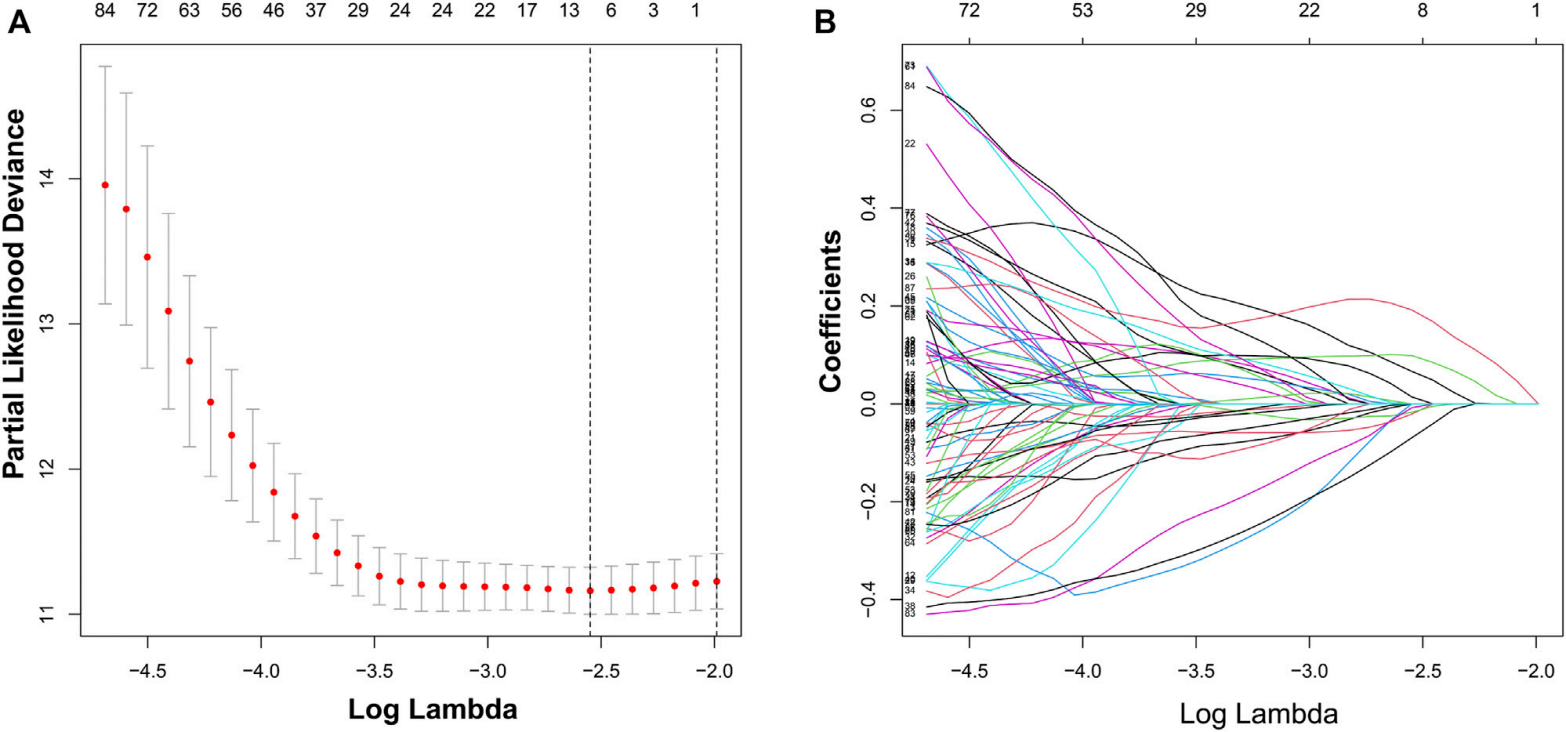
Identification of a risk signature by LASSO regression analysis. **(A)**, Cross-validation for tuning parameter selection in the proportional hazards model. **(B)**, LASSO coefficient spectrum of seven genes.

**FIGURE 4:**
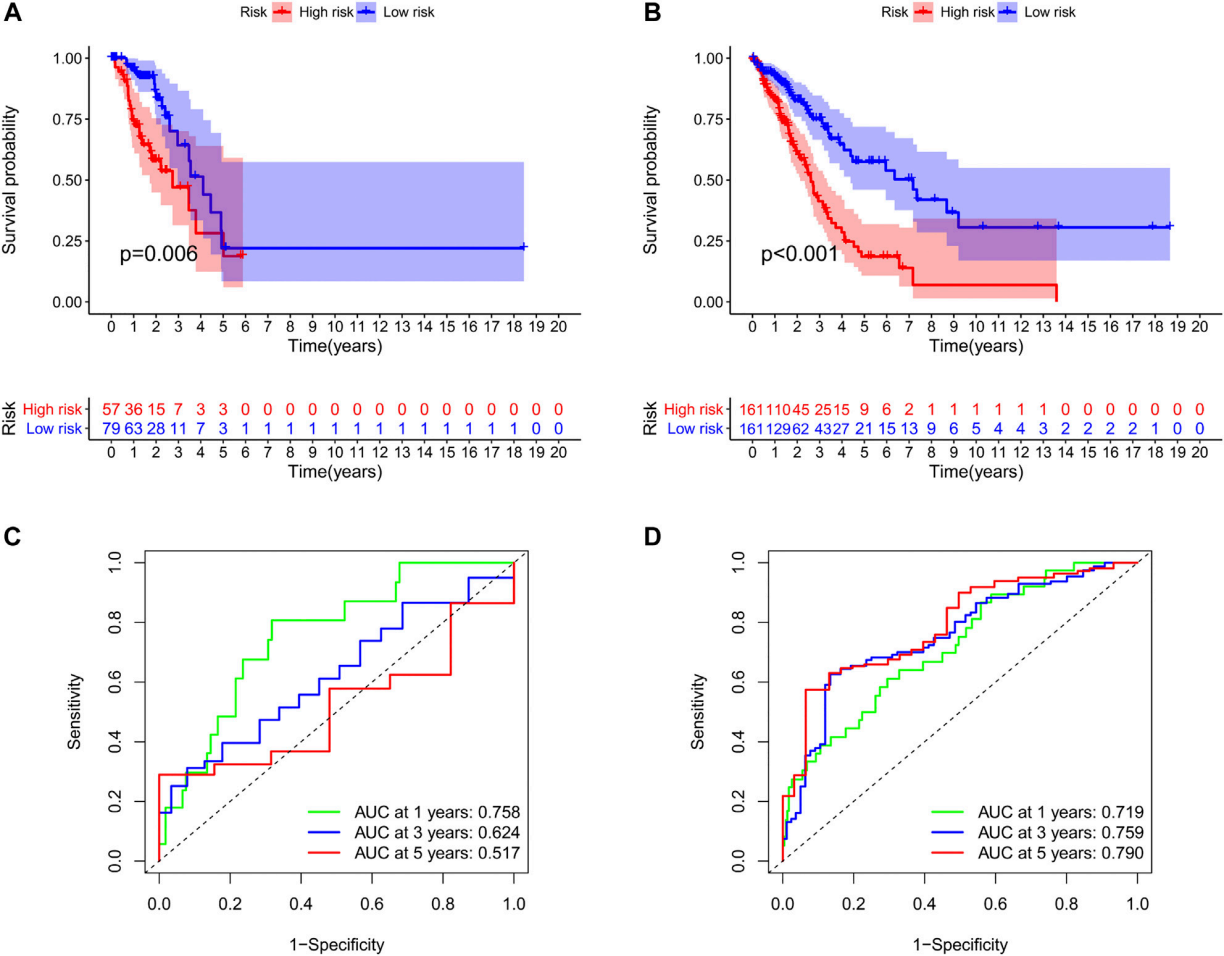
Survival analyses of two groups of patients. The survival was significantly different between the high-risk group and low-risk group in the training set **(A)** and the testing set **(B)**. The area under the receiver operating characteristic (ROC) curve in the training set **(C)** and the testing set **(D)**.

**FIGURE 5:**
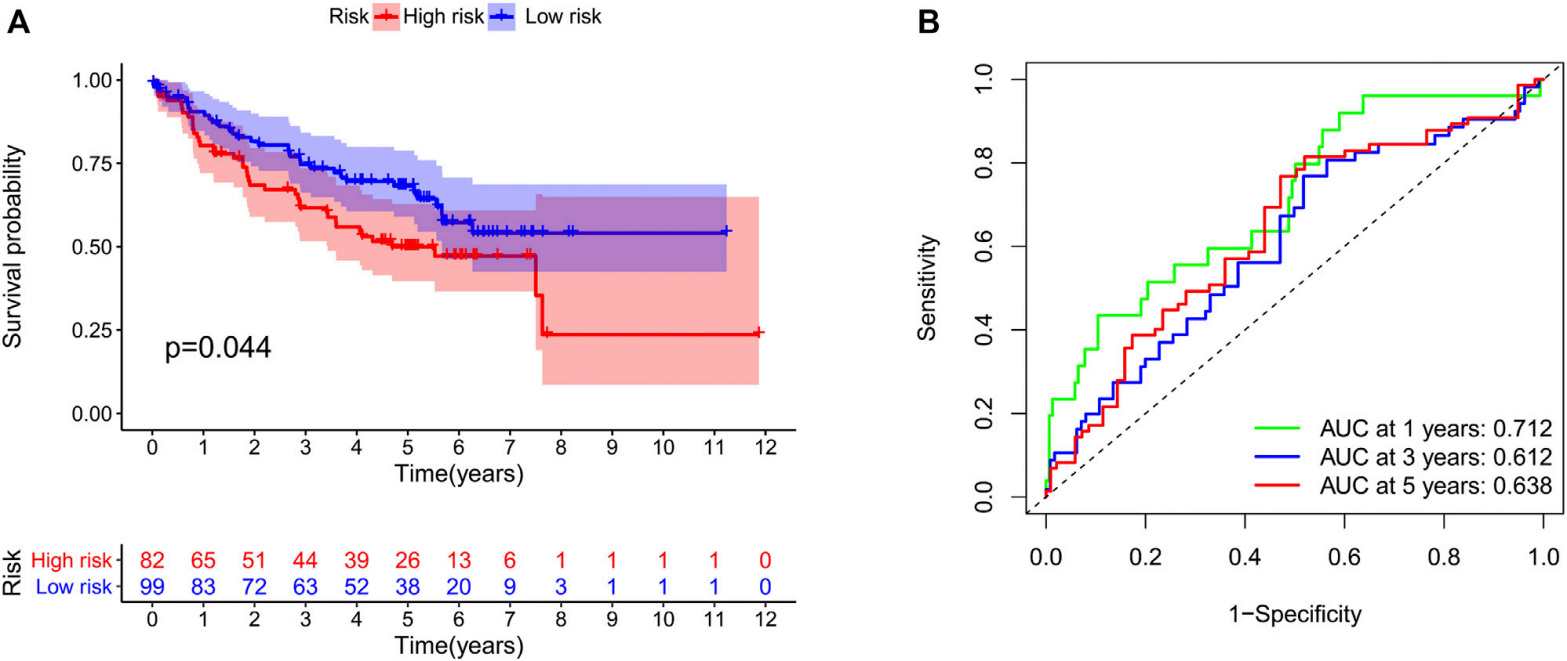
Validation of our risk model in the GSE50081 dataset. The survival time **(A)** and the area under the ROC curve **(B)** in the two groups of patients.

We identified 187 overall survival-associated genes from lung cancer patients in the TCGA cohort. To minimize the risk of overfitting, LASSO regression algorithm analysis was used to generate the best gene model (Figures 3A,B). Ultimately, a gene model with seven genes was created. These seven prognosis related genes play important roles in cancer progression. High-risk genes (C1QTNF6, LDHA, IGF2BP1) resulted in poor clinical outcomes by promoting cancer cell metabolism and proliferation. PLEK2 and FAM133A involved in tumor migration. In contrast, the tumor suppressor genes (BEX5, KLHL35) were correlated with longer survival time. We calculated the risk score for each patient according to the coefficient value of the seven genes. Subsequently, patients were classified into high-risk and low-risk groups based on the median risk score. We divided the patients from the TCGA cohort into the training set (*n* = 322) and the testing set (*n* = 136). There was no difference in clinical features between the testing group and the training group (Table 1). The sample ids was showed in Supplementary Material (Supplementary Data S1). First, we investigated the prognostic role of our model in the training set. A longer survival time was found in low-risk patients than in the high-risk training set (*p* = 0.006; Figure 4A) and in the testing set. To further evaluate the accuracy of the prognostic value of the risk score, time-dependent ROC curves were plotted (Figure 4C), and the area under the curve (AUC) at 1 year of overall survival (OS) was 0.758. Thereafter, the gene signature was tested for its prognostic performance in the independent TCGA testing set. Consistent with the training set, patients in different risk groups showed significantly different OS. Furthermore, the AUCs at 1 year, 3 years, and 5 years of OS were higher than 0.7 in the testing set (Figure 4D). To further validate our risk model in different platforms, we confirm our finding using data from GSE50081 (*n* = 181). High-risk patients had poorer survival times than low-risk patients (Figure 5A), yielding a 1-year AUC of 0.712 (Figure 5B). These results are similar to those observed in the TCGA training set and testing set. We have also verified the model using the data from GSE13213 (*n* = 117), confirming our previous finding (Supplementary Figure S3).

**TABLE 1:** The clinical characteristic of TCGA testing set and training set.

Characteristic	Total	Test group	Train group	*p*-value
Age				
<=65	224 (48.91%)	73 (53.68%)	151 (46.89%)	0.220
>65	234 (51.09%)	63 (46.32%)	171 (53.11%)	
Gender				
Female	250 (54.59%)	67 (49.26%)	183 (56.83%)	0.151
Male	208 (45.41%)	69 (50.74%)	139 (43.17%)	
Stage				
Stage I	247 (53.93%)	73 (53.68%)	174 (54.04%)	0.729
Stage II	110 (24.02%)	30 (22.06%)	80 (24.84%)	
Stage III	74 (16.16%)	23 (16.91%)	52 (16.15%)	
Stage IV	21 (4.59%)	10 (7.35%)	16 (4.97%)	
T stage				
T1	156 (34.06%)	39 (28.68%)	117 (36.35%)	0.327
T2	241 (52.62%)	75 (55.15%)	166 (51.55%)	
T3	39 (8.52%)	13 (9.55%)	26 (8.07%)	
T4	22 (4.80%)	9 (6.62%)	13 (3.03%)	
M stage				
M0	432 (94.32%)	126 (92.65%)	306 (95.03%)	0.376
M1	26 (5.68%)	10 (7.35%)	16 (4.97%)	
N stage				
N0	301 (65.72%)	85 (62.50%)	218 (67.70%)	0.122
N1	84 (18.34%)	22 (16.18%)	62 (19.25%)	
N2	65 (14.19%)	28 (20.59%)	41 (12.73%)	
N3	2 (0.44%)	1 (0.73%)	1 (0.32%)	
Risk score				
High risk	218 (47.60%)	57 (41.91%)	161 (50.00%)	0.125
Low risk	240 (52.40%)	79 (58.08%)	161 (50.00%)	

### Association with clinicopathologic factors

**FIGURE 6:**
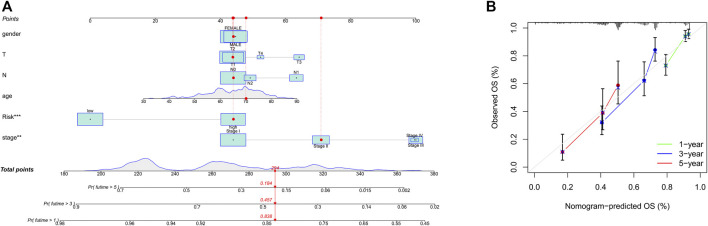
Prognosis prediction by the nomogram. **(A)**, A nomogram used to predict the overall survival. **(B)**, Calibration plots for survival.

**FIGURE 7:**
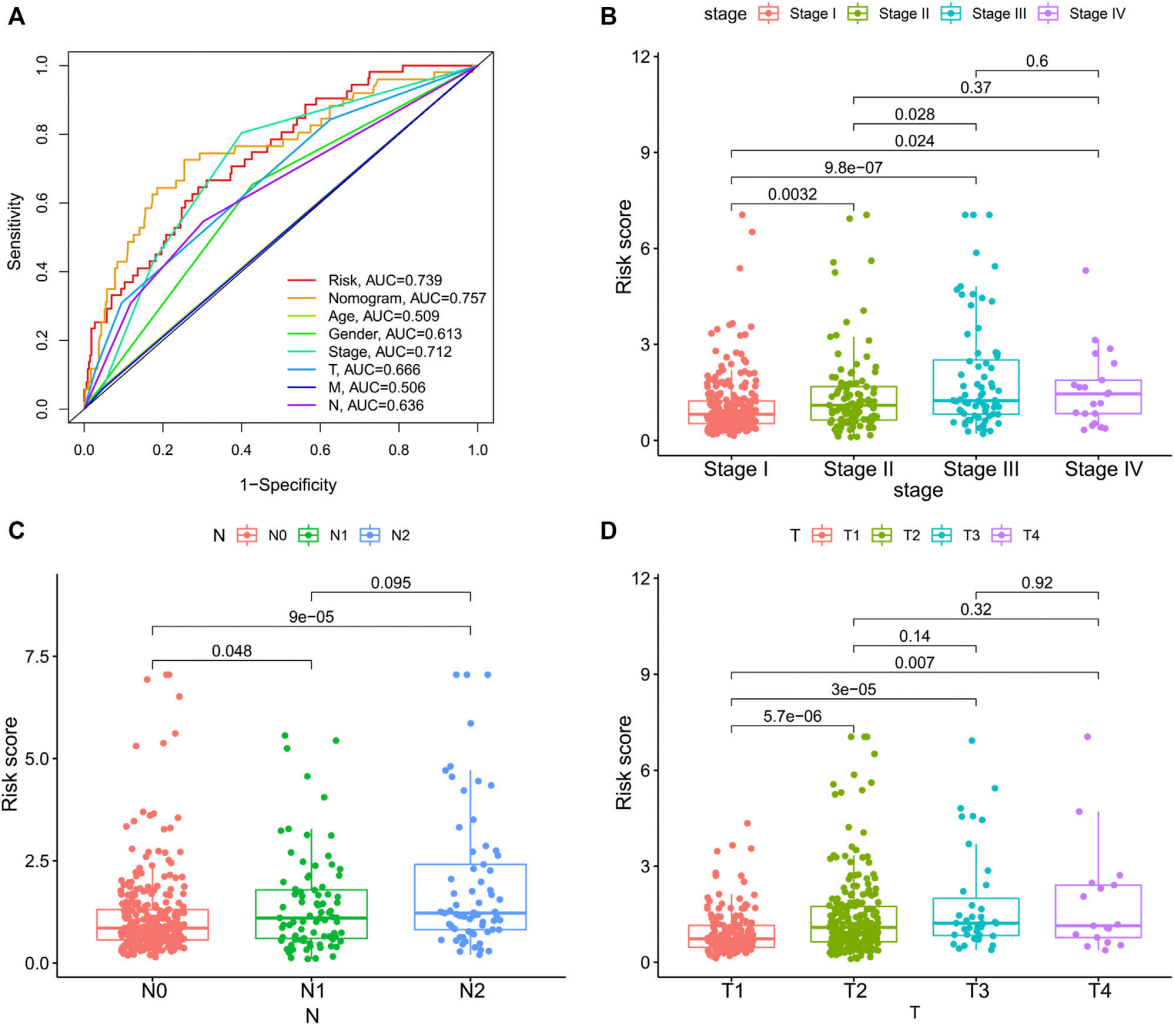
The associations between risk score and clinicopathologic factors. **(A)**, Multiindex ROC curve of risk score and other indicators. The association of risk score and stage **(B)**, N, lymphatic involvement **(C)**, and T, degree of tissue involvement **(D)**.

To integrate multiple risk factors, we used a nomogram to quantify the risk in lung cancer patients. Using the synthesis of seven gene signatures, a nomogram was constructed based on sex, age, stage, degree of tissue involvement (T), lymphatic involvement (N), and risk score to predict the probability of 1-, 3-, and 5-year OS (Figure 6A). Meanwhile, the calibration curve results showed that the predicted survival rate was closely related to the actual survival rate (Figure 6B). We then used a multi-index ROC curve to evaluate the accuracy of multiple risk indicators, where the nomogram and risk score showed superior accuracy (Figure 7A). In addition, we assessed the relationship between the risk score and clinicopathologic factors. We found that the risk score showed a good correlation with stage, T, and N (Figures 7B–D). Patients in the early stage tended to have lower risk scores than patients in the advanced stage. Additionally, in N and T, a higher risk score correlated with more malignant lung cancer.

### Predictive treatment response of the identified subgroups

**FIGURE 8:**
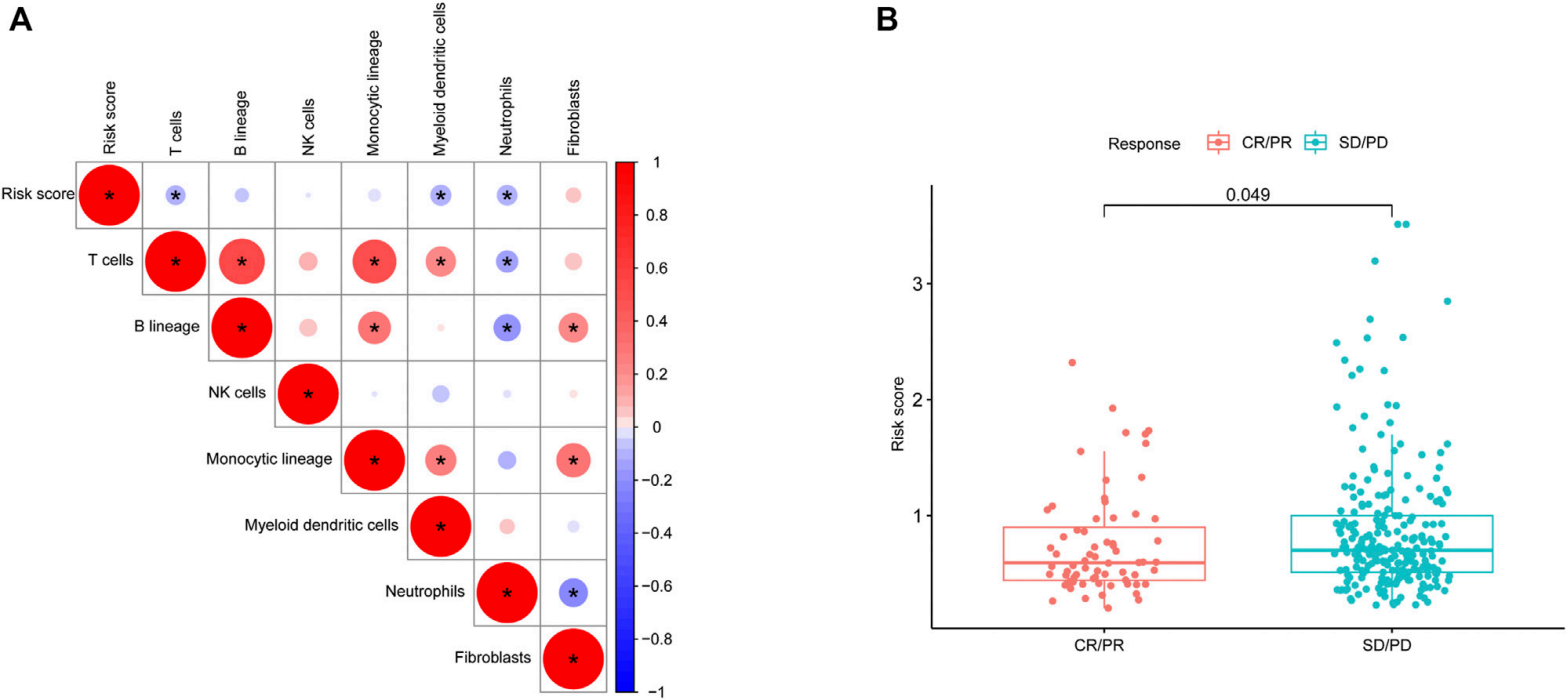
Microenvironmental characteristics of lung cancers. **(A)**, Correlation between the risk score and microenvironment-related cell types. **(B)**, Risk score distribution between patients with different treatment responses.

We studied the correlation between the risk score and microenvironment-related cell types and found that the patients with higher scores had less immune cell infiltration but more fibroblasts (Figure 8A). To further evaluate the predictive role of our gene model on treatment response, we compared the risk score between patients with different therapy responses. We defined patients with complete or partial response to treatment as having a satisfactory response, whereas lung cancer patients with a stable and progressive disease status were defined as having a poor response. Our results suggested that patients who were more sensitive to therapy had a lower risk score, indicating the predictive role of the risk score on treatment response (Figure 8B).

## Discussion

As stated by numerous studies, characterizing TME at high resolution to understand how tumor cells avoid surveillance to maintain proliferation and promote metastasis are of vital importance (Altorki et al., 2019). Genes that deeply involved in promoting tumor growth/TME appear to be capable of classifying NSCLC patients into two different prognostic subtypes. The high-risk subtype had higher proportion of cells that possessed regenerating ability and highly expressed Interferon-γ (IFN-γ), while the low-risk subtype had more inflammatory cells (the different immune subtypes identified by an extensive immunogenomic analysis of TCGA (Thorsson et al., 2018). Then, through LASSO regression analysis, we selected seven tumor microenvironment-associated genes to build a prediction model that could divide patients into two groups based on risk scores. The patients in the low-risk group seemed to show a better response to immunotherapy. Finally, we integrated the gene model score and clinicopathologic factors to construct a nomogram for predicting patient survival rates.

The TME consists of cellular components and the extracellular matrix (ECM). The cells include immune cells, cancer-associated fibroblasts, endothelial cells, and adipocytes (Ribeiro Franco et al., 2020). The TME plays an important role in the progression of tumors and the immune treatment response (Quail and Joyce, 2013). Tumor associated immune cells harbour infiltrating lymphoid and myeloid cells. Thymus dependent lymphocyte (T cells) are an important component of lymphocytes, which take part in cellular immunity. It is essential for tumor immunotherapy. Recently, Chimeric Antigen Receptor T-Cell (CAR-T) cell therapy was applied in clinical practice to counter the tumors. Some studies have shown that patients with more T cells in tumors seem to have better outcomes (Kishton et al., 2017; Mohanty et al., 2019). B cells are another main lymphocytes mediating humoral immunity by secreting immunoglobulins or promoting the T cell response (Tokunaga et al., 2019). Some studies have shown that higher numbers of B cells are associated with better prognosis (Eerola et al., 1999; Al-Shibli et al., 2008). For myeloid cell populations, Natural killer cells (NK cells) are also closely related to the tumor immune response (Morvan and Lanier, 2016). Past studies found that more infiltration of NK cells was associated with longer survival time in solid tumors (Nersesian et al., 2021). Neutrophils involved with the staging of oncogenesis, but the effect of neutrophil maturity on their antitumor activity or tumor promotion has not yet been elucidated (Mackey et al., 2019). Dendric cells (DC) take part in all procedures of the antitumor immune response by presenting antigens and synthetic peptides to activate T cells, consequently leading to an antitumor immune response. In this regard, a high level of DC is related to better progression-free survival time in NSCLC patients (Wang Y. et al., 2020). Currently, an increasing number of studies have focused on the function of tumor-associated macrophages (TAMs). TAMs can be classified into two categories: protumorigenic M2 macrophages and antitumorigenic M1 macrophages. TAMs were recruited by interleukin-17 of cancer cells, in return, it can specifically affect the aggressiveness of tumors through a variety of proteases. In addition to immune cells, fibroblasts also make up a major part of the TME. Cancer-associated fibroblasts (CAFs) can promote tumor growth and invasion *via* the secretion of tumor-related factors (Wang et al., 2009). For instance, CAFs can increase the expression of α-smooth muscle actin and downregulate the cell cycle gene p53 (Bar et al., 2009; Chatzistamou et al., 2011). Furthermore, alterations in the ECM also contribute to the development of tumors, such as protease-mediated matrix degradation and oxidative stress pathway activation (Wood et al., 2014). Considering the correlation between the TME and lung cancer prognosis, the related genes participating in the regulation of the TME and immune systems should be potential biomarkers for evaluations of prognosis and treatment response. In our study, we selected prognostic TME-associated genes to classify the two significantly different prognosis clusters and found that fewer immune cells but more CAFs infiltration in the tumor of high-risk patients. To predict clinical outcome accurately, a nomogram was constructed combining the risk score with clinicopathological factors.

We selected seven prognosis-related genes as our predictive signature. Similar to our study, Wang Q. et al. (2020) identified immune-related signatures of lung adenocarcinoma. Patients in two distinct subtypes were characterized by significantly different survival outcomes: TIDE score, programmed death-ligand 1 (PD-L1) expression, and tumor mutation burden (TMB). Song and Shang (2019) also built a model to predict the prognosis of NSCLC patients *via* immune-related genes, with a c-index of 0.723. Our prognostic model showed a similar rate compared with previous studies. However, our work focused on TME-related genes. The genes used in our risk prediction model are functional related not only to immune activity but also to the interaction between the tumor and microenvironment. The C1q/TNF-related protein (CTRP) family is involved in the body’s metabolism and immunity (Schäffler and Buechler, 2012). CTRPs play important roles in the development and progression of NSCLC by promoting metabolic disturbances, is one of independent risk factors for oncogenesis (Kong et al., 2021). PLEK2 (pleckstrin-2) participates in the epithelial-to-mesenchymal transition (EMT) progress of lung cancer cells, resulting in tumor invasion and metastasis (Wu et al., 2020). Lactate dehydrogenase A (LDHA) is an enzyme that catalyzes the interconversion of pyruvate and lactate. Some research has demonstrated that LDHA phosphorylation promotes cancer cell invasion and tumor metastasis (Jin et al., 2017; Feng et al., 2018). Additionally, IGF2BP1 (IGF2 mRNA binding proteins 1) was also reported to accelerate carcinogenesis (Huang et al., 2018). For protective genes, BEX5 is a member of the brain-expressed X-linked (BEX) family. A previous study demonstrated that BEX5 was associated with prognosis of NSCLC patients. Low expression levels of BEX5 indicate poor clinical outcomes (Zhang et al., 2019). Knockdown of KLHL35 (kelch-like family member 35) was also shown to increase tumor growth, providing direct functional evidence of tumor suppressor activity (Morris et al., 2011). In short, genes in our model are associated with tumor proliferation and partly take part in the regulation of the TME.

Nowdays, some researchers used patient-derived lung cancer organoids as *in vitro* models to predict drug responses. This cancer model could recapitulate the histological and genetic features of lung cancer and respond to drugs based on their genomic alterations. However, a critical limitation of the models is the lack of a cancer microenvironment (Kim et al., 2019). Considering the central role of the TME in the initiation and progression of lung cancer, we not only study the characteristics of immune cells, but also focused the genes which take part in regulating the TME. Even the gut microbiota may influence the cancer immune response. Recent study has analyzed the microbiota spectrum of lung cancer patients and established a gut microbial signature for the potential prediction of the early-stage lung cancer (Zheng et al., 2020). Comprehensive insights into the TME landscape in lung cancer may usher in a new era of cancer medicine.

In conclusion, this study classified NSCLC patients into two different prognosis clusters by TME-related genes. The two clusters seemed to display various immune subtype cells. A prognostic model constructed by the seven TME-related genes is presented that can independently predict prognosis of NCSLS patients. Our model was further validated by independent cohorts with data from GSE50081 and GSE13213. The high-risk group showed a worse immunotherapy response than the low-risk group. We also integrated the clinical factors and risk score to construct a nomogram for predicting prognosis. However, as our study was a retrospective analysis, statistical power was hampered by possible selection bias. Also, other clinical confounders (e.g., heterogeneous, populations pathological type) may influence the accuracy of the LASSO regression model. Further works are also needed to confirm the functions of these marker genes in lung cancer progression as well as their impact on patient survival.

## Data Availability

Publicly available datasets were analyzed in this study. This data can be found here: The Cancer Genome Atlas (TCGA) database and the Gene Expression Omnibus (GEO) database.

## References

[B1] Al-Shibli K. I. Donnem T. Al-Saad S. Persson M. Bremnes R. M. Busund L. T. (2008). Prognostic effect of epithelial and stromal lymphocyte infiltration in non-small cell lung cancer. Clin. Cancer Res. 14, 5220–5227. 10.1158/1078-0432.CCR-08-0133 PubMed Abstract | 10.1158/1078-0432.CCR-08-0133 | Google Scholar 18698040

[B2] Altorki N. K. Markowitz G. J. Gao D. Port J. L. Saxena A. Stiles B. (2019). The lung microenvironment: an important regulator of tumour growth and metastasis. Nat. Rev. Cancer 19, 9–31. 10.1038/s41568-018-0081-9 PubMed Abstract | 10.1038/s41568-018-0081-9 | Google Scholar 30532012 PMC6749995

[B3] Aran D. Hu Z. Butte A. J. (2017). xCell: digitally portraying the tissue cellular heterogeneity landscape. Genome Biol. 18, 220. 10.1186/s13059-017-1349-1 PubMed Abstract | 10.1186/s13059-017-1349-1 | Google Scholar 29141660 PMC5688663

[B4] Bar J. Feniger-Barish R. Lukashchuk N. Shaham H. Moskovits N. Goldfinger N. (2009). Cancer cells suppress p53 in adjacent fibroblasts. Oncogene 28, 933–936. 10.1038/onc.2008.445 PubMed Abstract | 10.1038/onc.2008.445 | Google Scholar 19060923 PMC2727601

[B5] Becht E. Giraldo N. A. Lacroix L. Buttard B. Elarouci N. Petitprez F. (2016). Estimating the population abundance of tissue-infiltrating immune and stromal cell populations using gene expression. Genome Biol. 17, 218. 10.1186/s13059-016-1070-5 PubMed Abstract | 10.1186/s13059-016-1070-5 | Google Scholar 27765066 PMC5073889

[B6] Chatzistamou I. Dioufa N. Trimis G. Sklavounou A. Kittas C. Kiaris H. (2011). p21/waf1 and smooth-muscle actin α expression in stromal fibroblasts of oral cancers. Cell. Oncol. 34, 483–488. 10.1007/s13402-011-0044-6 10.1007/s13402-011-0044-6 | Google Scholar PMC12994944 21559927

[B7] Chen Z. Fillmore C. M. Hammerman P. S. Kim C. F. Wong K. K. (2014). Non-small-cell lung cancers: a heterogeneous set of diseases. Nat. Rev. Cancer 14, 535–546. 10.1038/nrc3775 PubMed Abstract | 10.1038/nrc3775 | Google Scholar 25056707 PMC5712844

[B8] Chifman J. Pullikuth A. Chou J. W. Bedognetti D. Miller L. D. (2016). Conservation of immune gene signatures in solid tumors and prognostic implications. BMC Cancer 16, 911. 10.1186/s12885-016-2948-z PubMed Abstract | 10.1186/s12885-016-2948-z | Google Scholar 27871313 PMC5118876

[B9] Eerola A. K. Soini Y. Pääkkö P. (1999). Tumour infiltrating lymphocytes in relation to tumour angiogenesis, apoptosis and prognosis in patients with large cell lung carcinoma, Lung cancer. 26, 73–83. Amsterdam, Netherlands. 10.1016/s0169-5002(99)00072-0 PubMed Abstract | 10.1016/s0169-5002(99)00072-0 10568678

[B10] Feng Y. Xiong Y. Qiao T. Li X. Jia L. Han Y. (2018). Lactate dehydrogenase A: A key player in carcinogenesis and potential target in cancer therapy. Cancer Med. 7, 6124–6136. 10.1002/cam4.1820 PubMed Abstract | 10.1002/cam4.1820 | Google Scholar 30403008 PMC6308051

[B11] Gaujoux R. Seoighe C. (2010). A flexible R package for nonnegative matrix factorization. BMC Bioinforma. 11, 367. 10.1186/1471-2105-11-367 10.1186/1471-2105-11-367 | Google Scholar PMC2912887 20598126

[B12] Heagerty P. J. Zheng Y. (2005). Survival model predictive accuracy and ROC curves. Biometrics 61, 92–105. 10.1111/j.0006-341X.2005.030814.x PubMed Abstract | 10.1111/j.0006-341X.2005.030814.x | Google Scholar 15737082

[B13] Huang X. Zhang H. Guo X. Zhu Z. Cai H. Kong X. (2018). Insulin-like growth factor 2 mRNA-binding protein 1 (IGF2BP1) in cancer. J. Hematol. Oncol. 11, 88. 10.1186/s13045-018-0628-y PubMed Abstract | 10.1186/s13045-018-0628-y | Google Scholar 29954406 PMC6025799

[B14] Jin L. Chun J. Pan C. Alesi G. N. Li D. Magliocca K. R. (2017). Phosphorylation-mediated activation of LDHA promotes cancer cell invasion and tumour metastasis. Oncogene 36, 3797–3806. 10.1038/onc.2017.6 PubMed Abstract | 10.1038/onc.2017.6 | Google Scholar 28218905 PMC5501759

[B15] Kim H. Park H. (2007). Sparse non-negative matrix factorizations via alternating non-negativity-constrained least squares for microarray data analysis. Bioinforma. Oxf. Engl. 23, 1495–1502. 10.1093/bioinformatics/btm134 10.1093/bioinformatics/btm134 | Google Scholar 17483501

[B16] Kim M. Mun H. Sung C. O. Cho E. J. Jeon H.-J. Chun S.-M. (2019). Patient-derived lung cancer organoids as *in vitro* cancer models for therapeutic screening. Nat. Commun. 10, 3991. 10.1038/s41467-019-11867-6 PubMed Abstract | 10.1038/s41467-019-11867-6 | Google Scholar 31488816 PMC6728380

[B17] Kishton R. J. Sukumar M. Restifo N. P. (2017). Metabolic regulation of T cell longevity and function in tumor immunotherapy. Cell Metab. 26, 94–109. 10.1016/j.cmet.2017.06.016 PubMed Abstract | 10.1016/j.cmet.2017.06.016 | Google Scholar 28683298 PMC5543711

[B18] Kong M. Gao Y. Guo X. Xie Y. Yu Y. (2021). Role of the CTRP family in tumor development and progression. Oncol. Lett. 22, 723. 10.3892/ol.2021.12984 PubMed Abstract | 10.3892/ol.2021.12984 | Google Scholar 34429763 PMC8371956

[B19] Li B. Severson E. Pignon J. C. Zhao H. Li T. Novak J. (2016). Comprehensive analyses of tumor immunity: Implications for cancer immunotherapy. Genome Biol. 17, 174. 10.1186/s13059-016-1028-7 PubMed Abstract | 10.1186/s13059-016-1028-7 | Google Scholar 27549193 PMC4993001

[B20] Mackey J. B. G. Coffelt S. B. Carlin L. M. (2019). Neutrophil maturity in cancer. Front. Immunol. 10, 1912. 10.3389/fimmu.2019.01912 PubMed Abstract | 10.3389/fimmu.2019.01912 | Google Scholar 31474989 PMC6702268

[B21] Mahoney K. M. Rennert P. D. Freeman G. J. (2015). Combination cancer immunotherapy and new immunomodulatory targets. Nat. Rev. Drug Discov. 14, 561–584. 10.1038/nrd4591 PubMed Abstract | 10.1038/nrd4591 | Google Scholar 26228759

[B22] Mohanty R. Chowdhury C. R. Arega S. Sen P. Ganguly P. Ganguly N. (2019). CAR T cell therapy: A new era for cancer treatment (review). Oncol. Rep. 42, 2183–2195. 10.3892/or.2019.7335 PubMed Abstract | 10.3892/or.2019.7335 | Google Scholar 31578576

[B23] Morris M. R. Ricketts C. J. Gentle D. McRonald F. Carli N. Khalili H. (2011). Genome-wide methylation analysis identifies epigenetically inactivated candidate tumour suppressor genes in renal cell carcinoma. Oncogene 30, 1390–1401. 10.1038/onc.2010.525 PubMed Abstract | 10.1038/onc.2010.525 | Google Scholar 21132003

[B24] Morvan M. G. Lanier L. L. (2016). NK cells and cancer: you can teach innate cells new tricks. Nat. Rev. Cancer 16, 7–19. 10.1038/nrc.2015.5 PubMed Abstract | 10.1038/nrc.2015.5 | Google Scholar 26694935

[B25] Nersesian S. Schwartz S. L. Grantham S. R. MacLean L. K. Lee S. N. Pugh-Toole M. (2021). NK cell infiltration is associated with improved overall survival in solid cancers: A systematic review and meta-analysis. Transl. Oncol. 14, 100930. 10.1016/j.tranon.2020.100930 PubMed Abstract | 10.1016/j.tranon.2020.100930 | Google Scholar 33186888 PMC7670197

[B26] Newman A. M. Liu C. L. Green M. R. Gentles A. J. Feng W. Xu Y. (2015). Robust enumeration of cell subsets from tissue expression profiles. Nat. Methods 12, 453–457. 10.1038/nmeth.3337 PubMed Abstract | 10.1038/nmeth.3337 | Google Scholar 25822800 PMC4739640

[B27] Núñez E. Steyerberg E. W. Núñez J. (2011). Regression modeling strategies. Rev. Esp. Cardiol. 64, 501–507. 10.1016/j.recesp.2011.01.019 PubMed Abstract | 10.1016/j.recesp.2011.01.019 | Google Scholar 21531065

[B28] Quail D. F. Joyce J. A. (2013). Microenvironmental regulation of tumor progression and metastasis. Nat. Med. 19, 1423–1437. 10.1038/nm.3394 PubMed Abstract | 10.1038/nm.3394 | Google Scholar 24202395 PMC3954707

[B29] Ribas A. Wolchok J. D. (2018). Cancer immunotherapy using checkpoint blockade. Science 359, 1350–1355. 10.1126/science.aar4060 PubMed Abstract | 10.1126/science.aar4060 | Google Scholar 29567705 PMC7391259

[B30] Ribeiro Franco P. I. Rodrigues A. P. de Menezes L. B. Pacheco Miguel M. (2020). Tumor microenvironment components: Allies of cancer progression. Pathol. Res. Pract. 216, 152729. 10.1016/j.prp.2019.152729 PubMed Abstract | 10.1016/j.prp.2019.152729 | Google Scholar 31735322

[B31] Ritchie M. E. Phipson B. Wu D. Hu Y. Law C. W. Shi W. (2015). limma powers differential expression analyses for RNA-sequencing and microarray studies. Nucleic Acids Res. 43, e47. 10.1093/nar/gkv007 PubMed Abstract | 10.1093/nar/gkv007 | Google Scholar 25605792 PMC4402510

[B32] Rooney M. S. Shukla S. A. Wu C. J. Getz G. Hacohen N. (2015). Molecular and genetic properties of tumors associated with local immune cytolytic activity. Cell 160, 48–61. 10.1016/j.cell.2014.12.033 PubMed Abstract | 10.1016/j.cell.2014.12.033 | Google Scholar 25594174 PMC4856474

[B33] Schäffler A. Buechler C. (2012). CTRP family: linking immunity to metabolism. Trends Endocrinol. Metab. 23, 194–204. 10.1016/j.tem.2011.12.003 PubMed Abstract | 10.1016/j.tem.2011.12.003 | Google Scholar 22261190

[B34] Simon N. Friedman J. Hastie T. Tibshirani R. (2011). Regularization paths for cox's proportional hazards model via coordinate descent. J. Stat. Softw. 39, 1–13. 10.18637/jss.v039.i05 10.18637/jss.v039.i05 | Google Scholar PMC4824408 27065756

[B35] Song Q. Shang J. Yang Z. Zhang L. Zhang C. Chen J. (2019). Identification of an immune signature predicting prognosis risk of patients in lung adenocarcinoma. J. Transl. Med. 17, 70. 10.1186/s12967-019-1824-4 PubMed Abstract | 10.1186/s12967-019-1824-4 | Google Scholar 30832680 PMC6399972

[B36] Sung H. Ferlay J. Siegel R. L. Laversanne M. Soerjomataram I. Jemal A. (2021). Global cancer statistics 2020: GLOBOCAN estimates of incidence and mortality worldwide for 36 cancers in 185 countries. Ca. Cancer J. Clin. 71, 209–249. 10.3322/caac.21660 PubMed Abstract | 10.3322/caac.21660 | Google Scholar 33538338

[B37] Thorsson V. Gibbs D. L. Brown S. D. Wolf D. Bortone D. S. Ou Yang T. H. (2018). The immune landscape of cancer. Immunity 48, 812–830. e814. 10.1016/j.immuni.2018.03.023 PubMed Abstract | 10.1016/j.immuni.2018.03.023 | Google Scholar 29628290 PMC5982584

[B38] Tirosh I. Izar B. Prakadan S. M. Wadsworth M. H. 2nd Treacy D. Trombetta J. J. (2016). Dissecting the multicellular ecosystem of metastatic melanoma by single-cell RNA-seq. Science 352, 189–196. New York, NY. 10.1126/science.aad0501 PubMed Abstract | 10.1126/science.aad0501 | Google Scholar 27124452 PMC4944528

[B39] Tokunaga R. Naseem M. Lo J. H. Battaglin F. Soni S. Puccini A. (2019). B cell and B cell-related pathways for novel cancer treatments. Cancer Treat. Rev. 73, 10–19. 10.1016/j.ctrv.2018.12.001 PubMed Abstract | 10.1016/j.ctrv.2018.12.001 | Google Scholar 30551036 PMC7505165

[B40] Wang Q. Li M. Yang M. Yang Y. Song F. Zhang W. (2020a). Analysis of immune-related signatures of lung adenocarcinoma identified two distinct subtypes: Implications for immune checkpoint blockade therapy. Aging 12, 3312–3339. 10.18632/aging.102814 PubMed Abstract | 10.18632/aging.102814 | Google Scholar 32091408 PMC7066911

[B41] Wang W. Li Q. Yamada T. Matsumoto K. Matsumoto I. Oda M. (2009). Crosstalk to stromal fibroblasts induces resistance of lung cancer to epidermal growth factor receptor tyrosine kinase inhibitors. Clin. Cancer Res. 15, 6630–6638. 10.1158/1078-0432.CCR-09-1001 PubMed Abstract | 10.1158/1078-0432.CCR-09-1001 | Google Scholar 19843665

[B42] Wang Y. Zhao N. Wu Z. Pan N. Shen X. Liu T. (2020b). New insight on the correlation of metabolic status on (18)F-FDG PET/CT with immune marker expression in patients with non-small cell lung cancer. Eur. J. Nucl. Med. Mol. Imaging 47, 1127–1136. 10.1007/s00259-019-04500-7 10.1007/s00259-019-04500-7 | Google Scholar 31502013

[B43] Whitehead M. J. McCanney G. A. Willison H. J. Barnett S. C. (2019). MyelinJ: an ImageJ macro for high throughput analysis of myelinating cultures. Bioinforma. Oxf. Engl. 35, 4528–4530. 10.1093/bioinformatics/btz403 10.1093/bioinformatics/btz403 | Google Scholar PMC6821319 31095292

[B44] Wood S. L. Pernemalm M. Crosbie P. A. Whetton A. D. (2014). The role of the tumor-microenvironment in lung cancer-metastasis and its relationship to potential therapeutic targets. Cancer Treat. Rev. 40, 558–566. 10.1016/j.ctrv.2013.10.001 PubMed Abstract | 10.1016/j.ctrv.2013.10.001 | Google Scholar 24176790

[B45] Wu D. M. Deng S. H. Zhou J. Han R. Liu T. Zhang T. (2020). PLEK2 mediates metastasis and vascular invasion via the ubiquitin-dependent degradation of SHIP2 in non-small cell lung cancer. Int. J. Cancer 146, 2563–2575. 10.1002/ijc.32675 PubMed Abstract | 10.1002/ijc.32675 | Google Scholar 31498891

[B46] Zhang Z. H. Luan Z. Y. Han F. Chen H. Q. Liu W. B. Liu J. Y. (2019). Diagnostic and prognostic value of the BEX family in lung adenocarcinoma. Oncol. Lett. 18, 5523–5533. 10.3892/ol.2019.10905 PubMed Abstract | 10.3892/ol.2019.10905 | Google Scholar 31612060 PMC6781490

[B47] Zheng Y. Fang Z. Xue Y. Zhang J. Zhu J. Gao R. (2020). Specific gut microbiome signature predicts the early-stage lung cancer. Gut Microbes 11, 1030–1042. 10.1080/19490976.2020.1737487 PubMed Abstract | 10.1080/19490976.2020.1737487 | Google Scholar 32240032 PMC7524275

