# Supplementary material for: A tumor microenvironment gene set–Based prognostic signature for non-small-cell lung cancer
Source: Front Mol Biosci. 2022 Aug 10;9:849108. doi: 10.3389/fmolb.2022.849108 (PMC9400803; doi:10.3389/fmolb.2022.849108)
Supplement: Supplementary file 3 [file DataSheet1.docx]

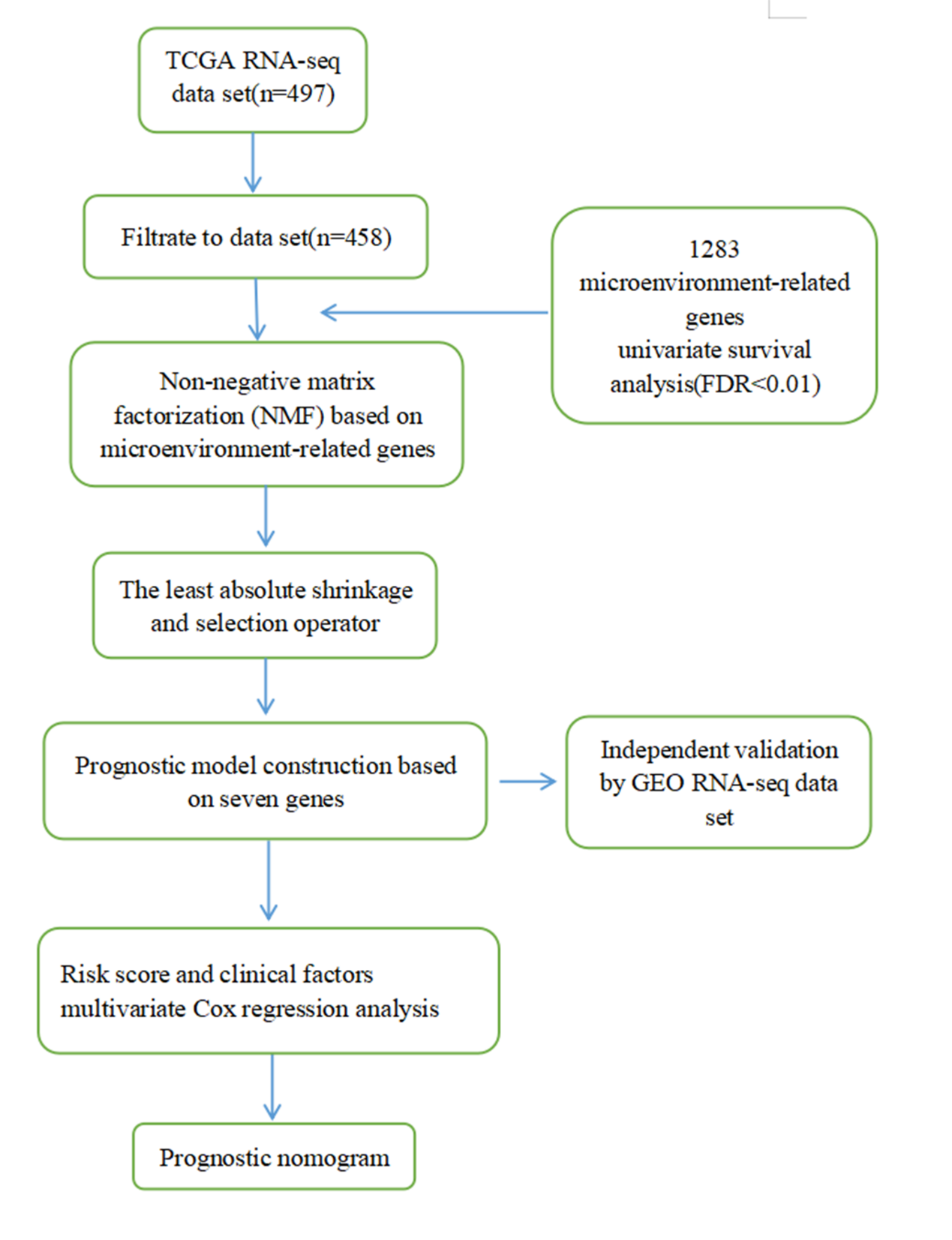


**Figure S1 Flow chart of the study**


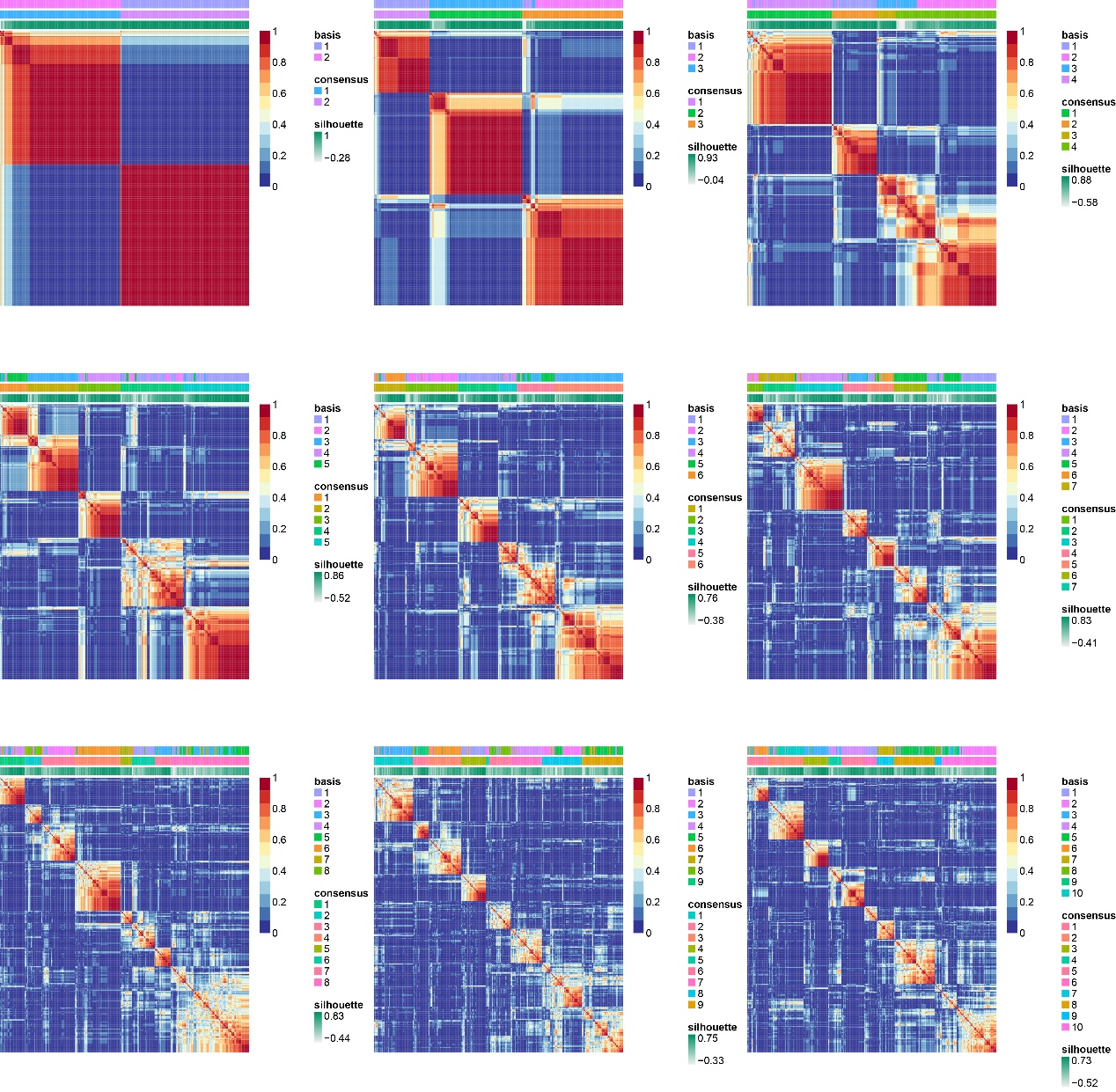


**Figure S2 Consensus matrix of NMF clustering for k from 2 to 10 in TCGA cohort.**


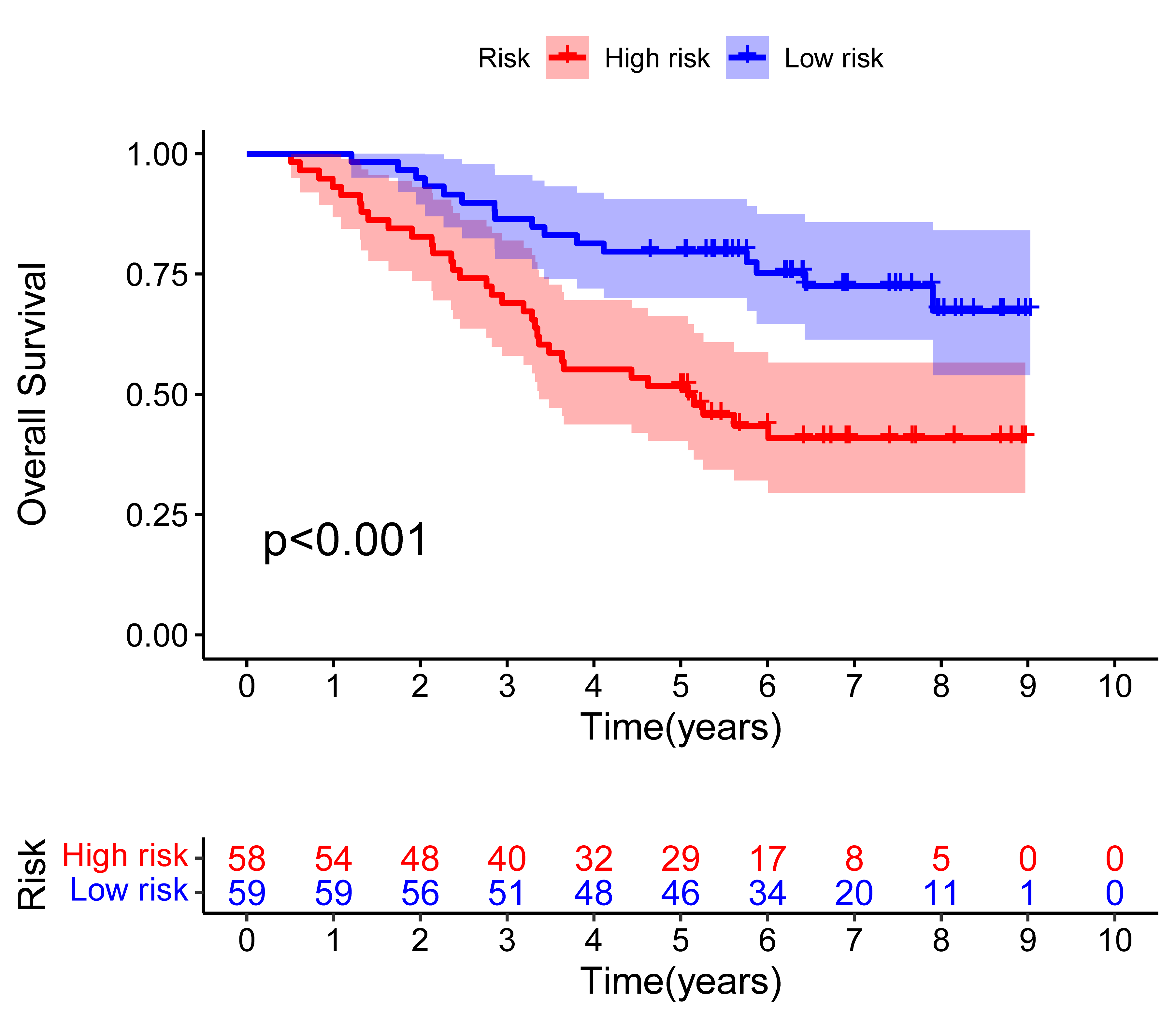


**Figure S3 Validation of our risk model in the GSE13213 dataset.**
